# Improved monitoring of clinical response in Systemic Lupus Erythematosus by longitudinal trend in soluble vascular cell adhesion molecule-1

**DOI:** 10.1186/s13075-015-0896-7

**Published:** 2016-01-08

**Authors:** Myles J. Lewis, Simon Vyse, Adrian M. Shields, Lu Zou, Munther Khamashta, Patrick A. Gordon, Costantino Pitzalis, Timothy J. Vyse, David P. D’Cruz

**Affiliations:** Centre for Experimental Medicine and Rheumatology, William Harvey Research Institute, Barts and The London School of Medicine and Dentistry, Queen Mary University of London, Charterhouse Square, London, EC1M 6BQ UK; Department of Medical and Molecular Genetics, King’s College London, London, SE1 9RT UK; Lupus Research Laboratory, The Rayne Institute, King’s College London, St Thomas’ Hospital, London, SE1 7EH UK; Rheumatology Department, King’s College Hospital, London, SE5 9RS UK

**Keywords:** Systemic lupus erythematosus, Soluble cell adhesion molecules, Vascular cell adhesion molecule-1, Biomarker, Memory B cells, Plasmablasts, Plasma cells, CD95, Complement C3, Anti-double-stranded DNA antibodies

## Abstract

**Background:**

To determine whether optimal use of serial measurements of serum levels of soluble cell adhesion molecules (CAM) can improve monitoring of disease activity in SLE.

**Methods:**

Serum levels of soluble CAM and conventional SLE biomarkers were measured in serial samples (n = 80) from 21 SLE patients during and after flare and correlated in longitudinal analysis with disease activity determined by ECLAM score. Blood samples from a second cohort of 34 SLE patients were subject to flow cytometry to correlate serum biomarkers with B cell subsets.

**Results:**

By adjusting for the baseline level (at the first visit), delta soluble vascular cell adhesion molecule-1 (sVCAM-1) showed stronger correlation with changes in ECLAM score and improved sensitivity and specificity for identifying SLE responders versus non-responders compared to conventional SLE biomarkers including anti-dsDNA antibody titre and complement C3. Multiple regression analysis identified delta sVCAM-1 as the best marker of SLE clinical response. sVCAM-1 levels were significantly correlated with CD95^+^CD27^+^ activated memory B cells, CD95^+^ plasmablasts and circulating plasma cell numbers in SLE patients.

**Conclusion:**

Subtracting a baseline level of sVCAM-1 for each individual substantially improved its utility as a biomarker. Delta sVCAM-1 was superior to conventional SLE biomarkers for monitoring changes in disease activity. This suggests that serial monitoring of serum sVCAM-1 trends should be considered in SLE patients to document responses to treatment. We hypothesise that the correlation between activated B cell subsets and circulating plasma cell numbers with soluble VCAM-1 serum levels in SLE may relate to the important role of VCAM-1 in B lymphocyte survival and maturation in bone marrow and secondary lymphoid tissues.

**Electronic supplementary material:**

The online version of this article (doi:10.1186/s13075-015-0896-7) contains supplementary material, which is available to authorized users.

## Background

Cell adhesion molecules (CAM) enable leukocyte adhesion and rolling along endothelial cell surfaces, and control migration of leukocytes into inflamed tissues [1]. With the aid of chemokines and chemoattractants, CAM regulate leukocyte circulation and allow lymphoid cells to home in on specific tissues or inflammatory sites. Adhesion molecules can be classified into three main groups: selectins, integrins and immunoglobulin supergene family (IGSF) groups. Vascular cell adhesion molecule-1 (VCAM-1) and intercellular adhesion molecule-1 (ICAM-1) are IGSF group members which are induced on endothelial cells in response to numerous inflammatory cytokines, including tumour necrosis factor (TNF) and interleukin (IL)-1, and bind integrin partners on leukocytes. E-selectin and P-selectin are also inducible on activated endothelium, with the latter additionally expressed on platelets. Soluble versions of VCAM-1, ICAM-1, E-selectin and P-selectin are shed from endothelial cell surfaces and are readily detectable in serum [2]. Soluble VCAM-1 (sVCAM-1) is the most abundant of the circulating CAM, and shows the greatest variation in serum level across a number of inflammatory diseases, with the highest levels observed in active systemic lupus erythematosus (SLE), renal allograft and septic shock [3, 4]. sVCAM-1 levels are elevated in several autoimmune rheumatic diseases including SLE and rheumatoid arthritis (RA) compared with healthy controls, but results for other CAM are conflicting [5–9]. sVCAM-1 levels have been shown to correlate with SLE disease activity in several studies [7–10], and generally appear to correlate better with disease activity than sICAM-1 or soluble E-selectin (sE-selectin). Urinary sVCAM-1 has been proposed as a biomarker in lupus nephritis [11–13]. High levels of sVCAM-1 have also been associated with severity of thrombosis in patients with antiphospholipid syndrome [14].

As well as being induced on activated endothelial cells, VCAM-1 is widely expressed on stromal cells in bone marrow and secondary lymphoid tissues, lymphatic endothelium and follicular dendritic cells (FDC) [15, 16]. Germinal centre dendritic cell (DC) expression of VCAM-1 is an important B-cell survival factor [17]. Bone marrow stromal cell expression of VCAM-1 regulates several physiological functions: retention/release of haemopoietic stem cell progenitors [18, 19]; pre-pro B-cell maturation [20]; and mature B-cell homing to bone marrow leading to long-lived plasma cell persistence [20–22]. VCAM-1 thus plays a global role in lymphocyte trafficking and homeostasis of lymphocyte development.

In the present study, we sought to refine the use of sVCAM-1 as a biomarker in SLE, and to determine whether longitudinal sampling of sVCAM-1 could be of clinical utility in addition to conventional serum markers of disease activity such as anti-double-stranded DNA (anti-dsDNA) antibody levels and complement C3 and C4 levels. We identified patients undergoing a flare of SLE and serially assayed serum levels of soluble CAM to determine whether soluble CAM either singly or in combination with conventional serum markers of disease activity could be used to more accurately monitor decreasing disease activity following treatment. Elevated sVCAM-1 levels during SLE flare have been previously assumed to be due to endothelial activation. We hypothesised that circulating VCAM-1 might also reflect accelerated B-cell maturation in secondary lymphoid tissue and/or abnormal turnover of lymphocyte progenitors and long-lived plasma cells in bone marrow. In a second cohort of SLE patients, we determined whether there was any association between soluble CAM levels and circulating B-cell subset numbers and B-cell activation.

## Methods

### Individuals

The study was approved by the UK National Research Ethics Service prior to the commencement of the study. All study participants provided written consent at the time of first sample collection. All SLE individuals fulfilled the American College of Rheumatology (ACR) criteria for classification of SLE [23]. For longitudinal analysis of serum biomarkers, 21 SLE patients (cohort 1) were identified who were undergoing a flare of SLE, defined as a significant increase in disease activity necessitating a change in treatment and in all cases consistent with the current consensus definition of lupus flare [24]. Repeated blood samples (*n* = 80) were obtained during regular follow-up visits as part of standard care (average time interval 4 months) with an aim of four samples per patient. SLE disease activity at each clinic visit was assessed by the European Consensus Lupus Activity Measure (ECLAM) [25]. Blood samples were obtained from a second, separate cohort of 34 SLE patients for comparison of serum markers and B-cell subsets measured by flow cytometry (see later). Demographics and active disease characteristics for both SLE cohorts are summarised in Table 1.

**Table 1:** Patient demographics and clinical characteristics at time of enrolment

Demographic variable	Cohort 1 (*n* = 21)	Cohort 2 (*n* = 34)
Female	19 (90 %)	31 (91 %)
Age, median (interquartile range)	41.0 (34.3–51.3)	44.5 (36.0–52.0)
Samples per patient, median (range)	4 (2–7)	1 (1–1)
Follow-up duration (months), median (interquartile range)	16.5 (12.0–21.3)	−
Active SLE disease features		
Cutaneous	10 (48 %)	19 (56 %)
Arthritis	4 (19 %)	13 (38 %)
Serositis	5 (24 %)	2 (6 %)
Renal	12 (57 %)	11 (32 %)
Neurologic	5 (24 %)	3 (9 %)
Haematologic	16 (76 %)	17 (50 %)
dsDNA-positive	15 (71 %)	19 (56 %)
Low C3/C4	13 (62 %)	15 (44 %)
Other SLE serology		
ANA-positive	17 (81 %)	33 (97 %)
Ro/La-positive	4 (19 %)	18 (53 %)
Sm/RNP-positive	3 (14 %)	22 (65 %)
Active treatment		
Initial prednisolone dose (mg/day), median (range)	15 (0-35)	5 (0-25)
Maintenance prednisolone dose (mg/day), median (range)	7.5 (0-30)	−
Mycophenolate mofetil	11 (52 %)	9 (26 %)
Azathioprine	10 (48 %)	6 (18 %)
Hydroxychloroquine	10 (48 %)	19 (56 %)
Methotrexate	0 (0 %)	6 (18 %)
Tacrolimus/everolimus	0 (0 %)	2 (6 %)
Previous treatment		
Intravenous cyclophosphamide	11 (52 %)	3 (9 %)
Intravenous immunoglobulin	2 (10 %)	0 (0 %)

*dsDNA* double-stranded DNA, *SLE* systemic lupus erythematosus, *ANA* anti-nuclear antibody , *Sm* anti-Smith, *RNP* anti-ribonucleoprotein

### Serum biomarker measurement

Serum levels of sVCAM-1, sICAM-1, sE-selectin and soluble P-selectin (sP-selectin) were analysed by sandwich enzyme-linked immunosorbent assay (ELISA) according to the manufacturer’s instructions (R&D Systems, Abingdon, UK). sVCAM-1 levels were measured in sera from four healthy control individuals (mean ± standard error (SE) of the mean 399.1 ± 105 ng/ml), comparable with previous studies [14]. The erythrocyte sedimentation rate (ESR), anti-dsDNA antibody levels, complement C3 and C4 levels, and C-reactive protein (CRP) were measured as part of routine clinical management of SLE patients. Anti-dsDNA antibody levels were screened by *Crithidia luciliae* immunofluorescence and assayed by radioimmunoassay (Farr assay). Complement C3 and C4 levels were assayed by nephelometry.

### Flow cytometry

Fresh peripheral blood mononuclear cells were isolated from blood obtained from 34 SLE individuals. Cells were stained with LIVE/DEAD Fixable Blue Dead cell stain (Invitrogen, Paisley, UK) to exclude dead cells, Fc receptor blocked (Human TruStain FcX; BioLegend, Oxford, UK) and surface stained using the following markers: IgD-BrilliantViolet(BV)421 (IA6-2), CD19-BV510 (HIB19), CD27-BV650 (O323), CD138-FITC or CD138-PE-Cy7 (MI15), CD24-PerCP-Cy5.5 (ML5), CD95-PE-Cy7 (DX2), CD38-APC (HB7) and CD20-APC-H7 (2H7) from BioLegend or BD. Cells were fixed with BD stabilising fixative reagent. Freshly stained cells were acquired on a 5 laser BD SORP LSRFortessa instrument. BD CS&T beads were used immediately prior to every sample run to maintain instrument consistency throughout the entire study. Data were analysed using FlowJo version 10 (Ashland, OR, USA).

### Statistical analysis

Statistical analysis was performed using SPSS statistics version 22 (IBM Corporation, Armonk, NY, USA) and R statistics package version 3.1 (R Foundation for Statistical Computing, Vienna, Austria). Biomarker performance was analysed by receiver operating characteristic (ROC) curve analysis, using the pROC package version 1.7.3 in R, and Youden’s index was used to select the optimal discriminatory threshold. A reduction in ECLAM score of 3 or more (∆ECLAM ≤ –3) was used to define “clinically meaningful” improvement in disease activity [26]. Delta parameters were calculated by subtracting the value on each individual’s first visit for each parameter. For analysis of ∆ECLAM, multiple linear regression was performed with stepwise selection based on Akaike information criteria (AIC), using a mixed-effects model to account for within-individual correlation because of repeated measures for each individual over time. The CD138^+^ plasma cell population size expressed as the percentage of B cells was analysed by multiple linear regression with stepwise selection and beta regression to account for the standard unit interval of this variable. Standardisation was applied to predictors in all models.

## Results

**Fig. 1:**
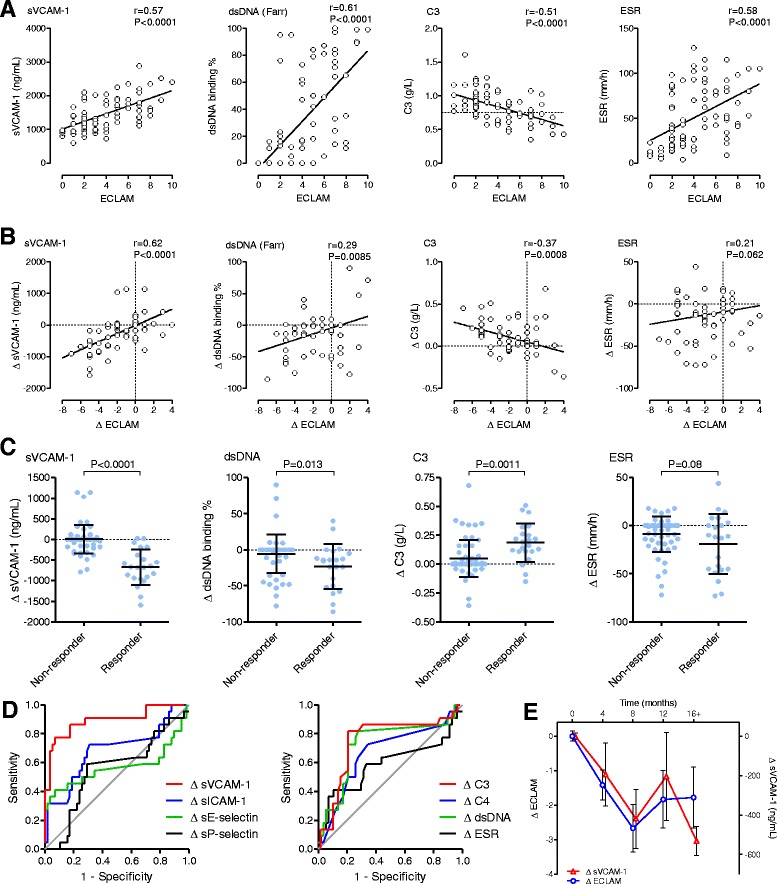
Subtraction of baseline improves biomarker ability of sVCAM-1 for tracking longitudinal changes in SLE disease activity. **a** sVCAM-1 assayed by ELISA and compared with SLE disease activity measured by ECLAM score. Correlation with ECLAM score is shown for conventional markers of disease activity: anti-dsDNA antibody titre by Farr radioimmunoassay, complement C3 level and ESR. *Dotted lines* represent laboratory lower limit for C3 to show individuals with hypocomplementaemia. Statistical analysis by Spearman correlation. **b** Correlation plots showing change in biomarker level against change in ECLAM score for sVCAM-1 compared with conventional serum biomarkers of SLE disease activity. Delta values for each parameter were calculated by subtracting the parameter value at the first visit for each individual. **c** Comparison of biomarker levels in SLE responders (∆ECLAM ≤ −3) versus nonresponders (∆ECLAM > −3) for ∆sVCAM-1, ∆anti-dsDNA titre, ∆C3 level and ∆ESR. Bars represent mean ± standard deviation. Statistical analysis by unpaired *t* test. **d** ROC curves for changing levels of serum biomarkers for detection of clinical response in SLE (∆ECLAM ≤ −3). **e** Graph showing interval time course of ∆sVCAM-1 levels compared with ∆ECLAM score. *dsDNA* double-stranded DNA, *ECLAM* European Consensus Lupus Activity Measure, *ESR* erythrocyte sedimentation rate, *sE-selectin* soluble E-selectin, *sICAM-1* soluble intercellular adhesion molecule-1, *sP-selectin* soluble P-selectin, *sVCAM-1* soluble vascular cell adhesion molecule-1

A total of 80 samples were assayed from 21 patients with a median of four samples per patient, covering a median follow-up duration of 16.5 months (interquartile range 12.0–21.3 months). Demographics for this first cohort of SLE patients are summarised in Table 1. Using Spearman rank correlation, the anti-dsDNA titre by radioimmunoassay (Farr) (*r* = 0.608, *P* = 2.2 × 10^−9^) and ESR (*r* = 0.584) showed the strongest correlations with ECLAM score, while sVCAM-1 levels (*r* = 0.571, *P* = 4.0 × 10^−8^) were more highly correlated than complement C3 (*r* = −0.510) and C4 (*r* = −0.416) levels (Fig. 1a, Table 2). sICAM-1, sE-selectin and sP-selectin were not significantly correlated with SLE disease activity. However, when using a mixed-effects model to account for repeated measures over time, sVCAM-1 showed a better fit (*P* = 2.4 × 10^−10^) than other parameters including conventional serum markers. ROC curve analysis was performed for each biomarker using a cutoff value for ECLAM score of greater than 3 (ECLAM >3) as a “clinically meaningful” measure of active versus inactive SLE [26]. sVCAM-1 was the only soluble CAM to be able to distinguish active lupus (area under curve (AUC) 0.796, 95 % CI 0.689 − 0.894), but performed less well than anti-dsDNA titre by both AUC and Youden index measures (Table 2). As expected, the anti-dsDNA titre was the most specific test of the conventional serum markers for identifying patients with high disease activity, while complement C3 and ESR were the most sensitive tests in this group of patients. This observation is consistent with previous reports which suggested that sVCAM-1 was superior to other soluble CAM in terms of correlation with disease activity [6, 7], but did not improve upon conventional markers for identifying active versus inactive disease [8].

**Table 2:** Performance characteristics of biomarkers to detect active SLE (ECLAM score >3)

	Spearman correlation		Mixed-effects	ROC curve analysis					
Biomarker	*r* value	*P* value	*P* value	AUC (95 % CI)	Youden index	Sensitivity (%)	Specificity (%)	PPV (%)	NPV (%)
sVCAM-1	0.571	4.0 × 10^−8^	2.4 × 10^−10^	0.796 (0.689 − 0.894)	0.546	82.1	72.5	74.4	80.6
sICAM-1	−0.067	NS	NS	0.536 (0.407 − 0.663)	0.175	20.0	97.5	88.9	54.9
sE-selectin	0.066	NS	1.3 × 10^−2^	0.496 (0.363 − 0.628)	0.200	47.5	72.5	63.3	58.0
sP-selectin	−0.111	NS	NS	0.535 (0.401 − 0.667)	0.200	67.5	52.5	58.7	61.8
ESR	0.584	1.6 × 10^−8^	7.1 × 10^−7^	0.818 (0.717 − 0.908)	0.543	82.5	71.8	75.0	80.0
dsDNA	0.608	2.2 × 10^−9^	1.1 × 10^−5^	0.832 (0.732 − 0.920)	0.600	75.0	85.0	83.3	77.3
C3	−0.510	1.0 × 10^−6^	1.8 × 10^−7^	0.804 (0.698 − 0.892)	0.500	82.5	67.5	71.7	79.4
C4	−0.416	1.3 × 10^−4^	2.2 × 10^−3^	0.744 (0.631 − 0.846)	0.450	77.5	67.5	70.5	75.0

*AUC* area under ROC curve, *CI* confidence interval, *dsDNA* double-stranded DNA, *ECLAM* European Consensus Lupus Activity Measure, *ESR* erythrocyte sedimentation rate, *NPV* negative predictive value, *NS* not significant, *PPV* positive predictive value, *r* Spearman correlation for each parameter against ECLAM, *ROC* receiver operating characteristic, *sE-selectin* soluble E-selectin, *sICAM-1* soluble intercellular adhesion molecule-1, *SLE* systemic lupus erythematosus, *sP-selectin* soluble P-selectin, *sVCAM-1* soluble vascular cell adhesion molecule-1

Since sVCAM-1 showed the strongest fit with ECLAM when a mixed-effects model was applied, we postulated that this could be explained by variability in baseline sVCAM-1 levels between individuals because of comorbidities such as atherosclerosis. To account for individual variation, changing levels of each biomarker (delta measurements) were calculated by subtracting baseline (first visit) values for each variable and correlating each biomarker with changing levels of ECLAM score (∆ECLAM) (Fig. 1b). Surprisingly ∆sVCAM-1 demonstrated the strongest correlation with ∆ECLAM (Spearman correlation, *r* = 0.622, *P* = 9.7 × 10^−10^), compared with ∆anti-dsDNA titre (*r* = 0.292, *P* = 0.0085), ∆C3 level (*r* = −0.367, *P* = 8.0 × 10^−4^) and ∆C4 level (*r* = −0.274, *P* = 0.014) (Table 3). Using a mixed-effects model incorporating time as a subject-specific effect, ∆sVCAM-1 (*P* = 3.85 × 10^−9^) still showed the best fit with ∆ECLAM compared with all other parameters. Responders were defined as a showing a “clinically meaningful” fall in ECLAM score of 3 or more (∆ECLAM score ≤ –3) compared with nonresponders (ΔECLAM > –3) [26]. Even a simple comparison of biomarker levels in responders versus nonresponders (Fig. 1c) showed that ∆sVCAM-1 demonstrated the widest delineation between responders and nonresponders (unpaired *t* test, *P* <0.0001) compared with dsDNA (*P* = 0.013) and C3 (*P* = 0.0011). ROC curve analysis confirmed that ∆sVCAM-1 (AUC 0.899, Youden index 0.703) had the best performance characteristics for identifying responders and nonresponders as determined by both ROC AUC and Youden index as compared with ∆complement C3 (AUC 0.772, Youden index 0.639), ∆dsDNA (AUC 0.731, Youden index 0.550) and all other biomarkers measured (Fig. 1d, Table 3). Based on the Youden index, ∆sVCAM-1 showed the highest sensitivity (86.4 %) of all parameters compared with a sensitivity of 81.8 % for both ∆C3 and ∆dsDNA. The optimal cutoff value for ∆sVCAM-1 corresponded to a reduction in sVCAM-1 level of 182 ng/ml. ∆sVCAM-1 also had a higher specificity (83.9 %) compared with both ∆C3 (82.1 %) and ∆dsDNA (73.2 %). Although ∆ESR (89.3 %) and ∆sE-selectin (92.9 %) had higher specificity than ∆sVCAM-1, both had very poor sensitivity (40.9 %). ∆sVCAM-1 showed the highest negative predictive value (94.0 %) of all parameters, and demonstrated a higher positive predictive value (67.9 %) compared with conventional biomarkers ∆C3, ∆C4, ∆dsDNA and ∆ESR for estimating improvement in the ECLAM score (Table 3). Furthermore, if specificity was more stringently fixed at 90 %, the sensitivity of ∆sVCAM-1 (77.3 %) was substantially superior to conventional biomarkers (∆ESR 40.9 %, ∆dsDNA 27.3 %, ∆C3 31.8 %) and other CAM. Plotting ∆sVCAM-1 and ∆ECLAM across 4-monthly time intervals showed that ∆sVCAM-1 effectively tracks ∆ECLAM over time (Fig. 1e), consistent with the results of the mixed-effects model. In an analysis of renal SLE patients, ∆sVCAM-1 showed a marginally stronger correlation with proteinuria (*r* = 0.27, *P* = 0.0041) compared with unadjusted sVCAM-1 (*r* = 0.22, *P* = 0.011) (Additional file 1: Figure S1A, B). ∆sVCAM-1 also showed significant correlation with ∆ECLAM in SLE individuals with negative dsDNA titres (*r* = 0.57, *P* <0.0001) (Additional file 1: Figure S1C) and in normocomplementaemic SLE individuals (*r* = 0.46, *P* = 0.0002) (Additional file 1: Figure S1D). This suggests that ∆sVCAM-1 has utility in a subset of SLE patients where standard SLE biomarkers such as dsDNA antibody titre and complement C3/C4 levels fail to reflect disease activity. However, we acknowledge that the present study is limited by numbers for these subgroup analyses, and a larger longitudinal study of ∆sVCAM-1 is required to confirm the usefulness of ∆sVCAM-1 in these SLE subgroups. Overall these results suggest that the ∆sVCAM-1 level was the best biomarker for forecasting changes in ECLAM score and identifying responders versus nonresponders.

**Table 3:** Performance characteristics of biomarkers to detect clinical response in SLE (reduction in ECLAM score of 3 or more)

	Spearman correlation		Mixed-effects	ROC curve analysis					
Biomarker	*r* value	*P* value	*P* value	AUC (95 % CI)	Youden index	Sensitivity (%)	Specificity (%)	PPV (%)	NPV (%)
∆sVCAM-1	0.622	9.7 × 10^−10^	3.9 × 10^−9^	0.899 (0.808 − 0.968)	0.703	86.4	83.9	67.9	94.0
∆sICAM-1	0.357	0.0012	3.4 × 10^−5^	0.688 (0.537 − 0.824)	0.426	72.7	69.9	48.5	86.7
∆sE-selectin	0.162	NS	1.0 × 10^−4^	0.569 (0.382 − 0.737)	0.338	40.9	92.9	69.2	80.0
∆sP-selectin	−0.092	NS	NS	0.541 (0.385 − 0.692)	0.287	59.1	69.6	43.3	81.2
∆ESR	0.211	0.062	8.3 × 10^−5^	0.586 (0.417 − 0.738)	0.302	40.9	89.3	60.0	79.4
∆dsDNA	0.292	0.0085	0.052	0.731 (0.581 − 0.860)	0.550	81.8	73.2	54.5	91.1
∆C3	−0.367	8.0 × 10^−4^	2.9 × 10^−4^	0.772 (0.634 − 0.890)	0.639	81.8	82.1	64.3	92.0
∆C4	−0.274	0.014	NS	0.689 (0.543− 0.821)	0.406	72.7	67.9	47.1	86.4

*AUC* area under ROC curve, *CI* confidence interval, *dsDNA* double-stranded DNA, *ECLAM* European Consensus Lupus Activity Measure, *ESR* erythrocyte sedimentation rate, *NPV* negative predictive value, *NS* not significant, *PPV* positive predictive value, *r* Spearman correlation for each parameter against ∆ECLAM, *ROC* receiver operating characteristic, *sE-selectin* soluble E-selectin, *sICAM-1* soluble intercellular adhesion molecule-1, *SLE* systemic lupus erythematosus, *sP-selectin* soluble P-selectin, *sVCAM-1* soluble vascular cell adhesion molecule-1

Further analysis was performed by multiple linear regression with stepwise selection using the following standardised variables: age, ∆C3, ∆C4, ∆dsDNA titre, ∆ESR, ∆sVCAM-1, ∆sICAM-1, ∆sE-selectin and ∆sP-selectin. A mixed-effects model was applied to take account of longitudinal measurements for each subject over time. As summarised in Table 4, both models identified two parameters, ∆sVCAM-1 (*P* = 8.6 × 10^−7^) and ∆C3 (*P* = 8.8 × 10^−4^), as being the optimal model for estimating improvement in disease activity measured by ∆ECLAM score (*P* = 6.0 × 10^−4^ for combined model compared with ∆sVCAM-1 alone). The final model yielded the following equation for estimating ΔECLAM using ΔsVCAM-1 (expressed in ng/ml) and ΔC3 (expressed in g/l):

**Table 4:** Multiple linear regression and mixed-effects model analyses for ∆ECLAM as a dependent variable

	Multiple linear regression		Mixed-effects model	
	Estimate (SE)	*P* value	Estimate (SE)	*P* value
∆sVCAM-1	1.126 (0.202)	3.5 × 10^−7^	1.280 (0.231)	8.6 × 10^−7^
∆C3	−0.764 (0.202)	3.0 × 10^−4^	−0.708 (0.201)	8.8 × 10^−4^

*ECLAM* European Consensus Lupus Activity Measure, *SE* standard error, *sVCAM-1* soluble vascular cell adhesion molecule-1

$$ \mathrm{Estimated}\ \Delta \mathrm{ECLAM} = \left[\Delta \mathrm{sVCAM}\hbox{-} 1\right] \times 0.00235\ \hbox{--}\ \left[\Delta \mathrm{C}3\right] \times 4.78\ \hbox{--}\ 0.537 $$

We tested this ∆sVCAM-1 and ∆C3 composite biomarker in our cohort to see whether it was an improvement compared with the use of ∆sVCAM-1 alone, and it made a marginal improvement to the AUC (0.927 95 % CI 0.872–0.983). However, at specificity levels above 80 % it did not significantly improve upon the sensitivity or specificity of ∆sVCAM-1 alone. Although in practice the ∆sVCAM-1 and ∆C3 composite biomarker did not offer much improvement over the use of ∆sVCAM-1 alone, these results confirm that even with inclusion of other parameters ∆sVCAM-1 is still the strongest estimator of disease activity response as measured by change in ECLAM score.

**Fig. 2:**
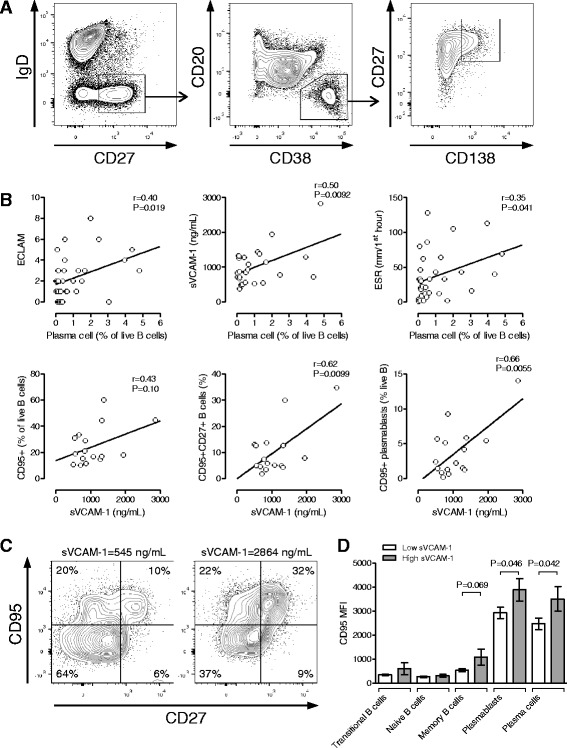
Serum levels of VCAM-1 correlate with circulating plasma cell numbers in SLE. B-cell subsets were assessed by 10-colour flow cytometry of fresh blood samples from SLE patients. **a** Representative gating plots showing plasma cell gating (CD19^mid^CD20^−^CD27^hi^CD38^hi^IgD^−^CD138^hi^). **b** Percentage of circulating plasma cells compared with SLE disease activity assessed by ECLAM score, serum sVCAM-1 level and ESR. sVCAM-1 levels were correlated against numbers of CD95^+^ activated B cells, CD95^+^CD27^+^ memory B cells and CD95^+^ plasmablasts (CD19^mid^CD20^−^CD27^hi^CD38^hi^IgD^−^). sVCAM-1 levels were measured by ELISA. Statistical analysis by Pearson correlation. **c** Representative plots of CD95 and CD27 expression in B cells from SLE patients with low and high serum sVCAM-1 levels. **d** CD95 mean fluorescence intensity (*MFI*) in B-cell subsets in SLE patients grouped into low and high sVCAM-1 levels. Statistical analysis by unpaired *t* test. *ECLAM* European Consensus Lupus Activity Measure, *ESR* erythrocyte sedimentation rate, *sVCAM-1* soluble vascular adhesion molecule-1

We postulated that the strong ability of ∆sVCAM-1 as a biomarker of clinical response in SLE, compared with sICAM-1 or sE-selectin which are also shed from activated endothelial cells, or CRP which is a known marker of vascular inflammation [27], suggested that circulating VCAM-1 levels could reflect factors other than endothelial inflammation, such as aberrant lymphocyte homeostasis in active SLE. B-cell subset numbers measured by 10-colour flow cytometry on fresh blood samples from a second cohort of 34 SLE patients were compared with the serum level of sVCAM-1 and conventional SLE serum markers (gating strategy shown in Fig. 2a). The numbers of total B cells, transitional B cells and naïve B cells were not significantly correlated with levels of sVCAM-1 (data not shown). However, the CD19^mid^CD20^−^CD27^hi^CD38^hi^IgD^−^CD138^hi^ circulating plasma cell number showed correlation with sVCAM-1 (Pearson’s correlation, *r* = 0.50, *P* = 0.0092) and ECLAM score (*r* = 0.40, *P* = 0.019), weak correlation with the ESR (*r* = 0.35, *P* = 0.041) (Fig. 2b) and a nonsignificant trend with dsDNA titre (*r* = 0.33, *P* = 0.088). This result is consistent with previous reports showing correlation between plasma cell number and SLE disease activity [28]. We observed significant correlation between serum sVCAM-1 levels and activated B-cell subsets including CD95^+^CD27^+^ memory B cells (*r* = 0.62, *P* = 0.0099) and CD95^+^ plasmablasts (CD19^mid^CD20^−^CD27^hi^CD38^hi^IgD^−^) (*r* = 0.66, P *=* 0.0055) (Fig. 2b, c), consistent with the involvement of these subsets of activated mature B cells in plasma cell generation. We also observed higher CD95 expression in plasmablasts (unpaired *t* test, *P* = 0.046) and plasma cells (*P* = 0.042) of SLE patients with high sVCAM-1 levels compared with low sVCAM-1 SLE patients (Fig. 2d). sVCAM-1 levels also correlated with CD95^+^ plasma cells (*r* = 0.61, *P* = 0.012), primarily because the vast majority of plasma cells were CD95^+^. Although the CD95^+^CD27^−^ memory B-cell subset has been shown to be increased in SLE in association with disease flares [29], we did not observe any correlation between the CD95^+^CD27^−^ subset of memory B cells and sVCAM-1 in this small cohort.

Multiple linear regression with beta regression was performed using the following standardised variables: sVCAM-1, ECLAM score, dsDNA, C3, C4, ESR and CRP. Beta regression was applied because plasma cell numbers (expressed as a percentage of live B cells) assume values within the standard unit interval. Stepwise selection identified a three-variable model including ESR, sVCAM-1 and ECLAM score. Both ESR (estimate (SE) –0.475 (0.226), *P*(>|*z*|) = 0.035) and sVCAM-1 (estimate (SE) 0.314 (0.153), *P* = 0.040) reached statistical significance by beta regression, while ECLAM showed borderline statistical significance (estimate (SE) 0.346 (0.190), *P* = 0.068). These results show that the correlation between sVCAM-1 levels and plasma cell numbers remains statistically significant even after adjustment for the known association between sVCAM-1 and ECLAM score.

## Discussion

These results recapitulate previous studies which have shown that serum levels of sVCAM-1 track disease activity in SLE [6, 7]. However consistent with these previous studies, we found that absolute levels of sVCAM-1 were less useful as a diagnostic tool for identifying SLE patients with active disease than other conventional markers such as dsDNA antibody titre and C3 levels [8], presumably due to intrinsic variability in sVCAM-1 levels between individuals. The key novel finding in our study is that subtracting a baseline level of sVCAM-1 significantly improved its biomarker abilities. We observed that the trend in changing levels of sVCAM-1 is a stronger marker of clinical response as measured by reduction in ECLAM score, compared with conventional serum markers of SLE disease activity (anti-dsDNA antibody, complement C3 and C4 levels, ESR), as well as other soluble CAM (sICAM-1, sE-selectin, sP-selectin). Our data show that a reduction in sVCAM-1 level of 182 ng/ml showed the highest sensitivity (86.4 %) for identifying responders, defined as showing a “clinically meaningful” fall in ECLAM score of −3, with comparably high specificity (83.9 %). Multiple linear regression analysis showed that ∆sVCAM-1 and ∆C3 are the two best markers of clinical response (as measured by ∆ECLAM), and confirmed that ∆sVCAM-1 is the best single marker of clinical response in SLE. We tested a composite score of both ∆sVCAM-1 and ∆C3 and found that it showed better correlation with ∆ECLAM, but did not usefully improve sensitivity and specificity compared with using ∆sVCAM-1 alone. We cannot rule out that in certain circumstances monitoring trends in serum levels of both sVCAM-1 and C3 may be a better method of tracking flares and response to therapy in SLE, although this will need to be determined in a larger study, to see whether particular subsets of patients benefit from a composite score including both parameters.

In the past, increased serum levels of sVCAM-1 have been attributed solely to endothelial activation and a large number of studies have examined serum levels of CAM in vascular disease and other conditions. In general, these studies show that sICAM-1 is a better predictor of future cardiovascular events, and mostly show that serum concentrations of sVCAM-1 and sICAM-1 are only modestly increased in coronary artery disease and diseases associated with endothelial dysfunction. This is consistent with evidence that sICAM-1 levels more closely reflect levels of systemic endothelial dysfunction than sVCAM-1 [30]. In contrast, levels of sVCAM-1 observed in patients with high SLE disease activity are among the highest levels of sVCAM-1 observed, showing levels comparable with patients with septic shock [4]. Our observation that sVCAM-1 levels track SLE disease activity far more strongly than sICAM-1, sE-selectin or sP-selectin suggests that fluctuations in sVCAM-1 levels reflect other physiological processes distinct from endothelial activation.

Surprisingly little is known of the physiological conditions required for the release of soluble CAM. VCAM-1, ICAM-1 and E-selectin are not constitutively expressed on the surface of endothelial cells, but are upregulated in response to stimulation such as by TNF, and during this process sVCAM-1, sICAM-1 and sE-selectin are shed from the cell membrane [2]. CAM expression is necessary for leukocyte adherence and migration into inflamed tissues, but shedding of VCAM-1 is not necessary for leukocyte detachment from endothelial surfaces during transendothelial migration, since this process is facilitated by calpain-mediated integrin cleavage [31]. This fits with lupus mouse model studies which show that although local VCAM-1 expression is elevated in lupus-affected organs [32], ICAM-1 is more critical for transendothelial migration of leukocytes into inflamed tissues [33, 34]. Cleavage of VCAM-1 from activated endothelial cells is predominantly mediated by TNFα converting enzyme (TACE, ADAM17) and regulated by TIMP-3 [35, 36], although other ADAM family metalloproteinases may also be involved [37]. Another situation in which VCAM-1 is deliberately shed is during granulocyte-colony stimulated factor (G-CSF)-induced mobilisation of haemopoietic stem cell progenitors from bone marrow [18, 19]. Notably the rise in serum sVCAM-1 induced by G-CSF is of a similar magnitude to that of active SLE patients.

VCAM-1 plays an important role at several key points of lymphocyte development because of its widespread expression on stromal cells in secondary lymphoid tissues [15, 16]. VCAM-1 is highly expressed on activated FDC [38, 39] where it serves two key purposes: VCAM-1 rescues B cells from apoptosis [17], and VCAM-1 is a prerequisite for DC to B cell immunological synapse during B-cell receptor antigen presentation [40]. VCAM-1 is highly expressed on CXCL12^+^ (SDF-1) bone marrow stromal cells and is required for both early pre-pro B-cell development as well as long-lived plasma cell retention in bone marrow [20]. Conditional deficiency of VCAM-1 in mice blocks B-cell maturation, resulting in increased circulating immature B cells, showing that VCAM-1 plays an important role in homing of mature B cells to bone marrow [21, 22]. It is unknown whether sVCAM-1 is shed during any of the numerous cell–cell interactions between lymphocytes and DC or other stromal cells which involve VCAM-1.

In the second part of our study, flow cytometric measurement of B-cell subsets in a second cohort of SLE patients revealed that sVCAM-1 levels were correlated with circulating plasma cell numbers, confirmed by multiple regression analysis. High sVCAM-1 levels were associated with increased expression of the activation marker CD95 in SLE plasmablasts and plasma cells. sVCAM-1 levels were correlated with activated B-cell subsets including CD95^+^CD27^+^ memory B cells and CD95^+^ plasmablasts, consistent with the importance of these B-cell subsets in aberrant plasma cell development in SLE [29]. sVCAM-1 could be derived from multiple sources: activated endothelium, bone marrow stromal cells, lymphatic endothelium and activated DCs. However, the relative quantities of sVCAM-1 shed from each of these sources are unknown. It is plausible that elevated serum levels of VCAM-1 in SLE patients not only reflect widespread endothelial activation in response to SLE-mediated tissue damage, but may reflect other active immune processes such as haematopoietic cell turnover in bone marrow, leukocyte trafficking via the lymphatic system, B-cell maturation by germinal centre FDC and homing/maintenance of long-lived plasma cells to bone marrow. This fits with our data indicating that sVCAM-1 showed a much stronger correlation with fluctuating SLE disease activity compared with sICAM-1 and sE-selectin, whose levels reflect systemic endothelial activation. sVCAM-1 may be released during times of increased lymphocyte turnover as a direct reflection of immunological changes during active SLE, which might explain the association between increased activated CD95^+^CD27^+^ memory B cells, CD95^+^ plasmablasts and circulating plasma cell numbers in SLE with higher serum levels of sVCAM-1.

## Conclusions

Our study has shown that serial monitoring of the trend in sVCAM-1 levels compared with a baseline reference level for each individual may be a useful additional biomarker for monitoring disease activity in SLE, and in this study population was superior to complement C3, dsDNA titre and ESR for identifying clinical response. This warrants further validation in larger SLE cohorts. The limitations of current markers of SLE disease activity are well recognised [41]: a significant proportion of SLE patients are either persistently negative for anti-dsDNA antibodies or do not develop hypocomplementaemia. Hence there is a major need for additional biomarkers which can improve monitoring of SLE disease activity and response to therapy. Our data suggest that serial measurements of ∆sVCAM-1 could lead to improved monitoring of clinical response to therapy in SLE and may be a useful novel outcome measure in future clinical trials of SLE.

## Additional file

Additional file 1: Figure S1. Showing analysis of ∆sVCAM-1 levels in SLE subgroups. Plots showing correlation of **A** unadjusted sVCAM-1 and **B** ∆sVCAM-1 levels with proteinuria levels in individuals with lupus nephritis. Correlation of ∆sVCAM-1 with change in SLE disease activity measured by ∆ECLAM in **C** SLE individuals with negative dsDNA titres and **D** normocomplementaemic SLE individuals. (PDF 36 kb)

## Abbreviations

AIC: Akaike information criteria
ACR: American College of Rheumatology
AUC: Area under curve
CAM: Cell adhesion molecules
CRP: C-reactive protein
DC: Dendritic cell
dsDNA: Double-stranded DNA
ECLAM: European Consensus Lupus Activity Measure
ELISA: Enzyme-linked immunosorbent assay
ESR: Erythrocyte sedimentation rate
FDC: Follicular dendritic cells
G-CSF: Granulocyte-colony stimulated factor
ICAM-1: Intercellular adhesion molecule-1
IGSF: Immunoglobulin supergene family
IL: Interleukin
RA: Rheumatoid arthritis
ROC: Receiver operating characteristic
SE: Standard error
sE-selectin: Soluble E-selectin
sICAM-1: Soluble intercellular adhesion molecule-1
SLE: Systemic lupus erythematosus
sP-selectin: Soluble P-selectin
sVCAM-1: Soluble vascular cell adhesion molecule-1
TNF: Tumour necrosis factor
VCAM-1: Vascular cell adhesion molecule-1

## References

[1] Springer TA (1994). Traffic signals for lymphocyte recirculation and leukocyte emigration: the multistep paradigm. Cell..

[2] Pigott R, Dillon LP, Hemingway IH, Gearing AJ (1992). Soluble forms of E-selectin, ICAM-1 and VCAM-1 are present in the supernatants of cytokine activated cultured endothelial cells. Biochem Biophys Res Commun..

[3] Gearing AJ, Newman W (1993). Circulating adhesion molecules in disease. Immunol Today..

[4] Zonneveld R, Martinelli R, Shapiro NI, Kuijpers TW, Plotz FB, Carman CV (2014). Soluble adhesion molecules as markers for sepsis and the potential pathophysiological discrepancy in neonates, children and adults. Crit Care..

[5] Mason JC, Kapahi P, Haskard DO (1993). Detection of increased levels of circulating intercellular adhesion molecule 1 in some patients with rheumatoid arthritis but not in patients with systemic lupus erythematosus. Lack of correlation with levels of circulating vascular cell adhesion molecule 1. Arthritis Rheum.

[6] Janssen BA, Luqmani RA, Gordon C, Hemingway IH, Bacon PA, Gearing AJ (1994). Correlation of blood levels of soluble vascular cell adhesion molecule-1 with disease activity in systemic lupus erythematosus and vasculitis. Br J Rheumatol..

[7] Spronk PE, Bootsma H, Huitema MG, Limburg PC, Kallenberg CG (1994). Levels of soluble VCAM-1, soluble ICAM-1, and soluble E-selectin during disease exacerbations in patients with systemic lupus erythematosus (SLE); a long term prospective study. Clin Exp Immunol..

[8] Horak P, Scudla V, Hermanovo Z, Pospisil Z, Faltynek L, Budikova M (2001). Clinical utility of selected disease activity markers in patients with systemic lupus erythematosus. Clin Rheumatol..

[9] Ho CY, Wong CK, Li EK, Tam LS, Lam CW (2003). Elevated plasma concentrations of nitric oxide, soluble thrombomodulin and soluble vascular cell adhesion molecule-1 in patients with systemic lupus erythematosus. Rheumatology (Oxford).

[10] Ikeda Y, Fujimoto T, Ameno M, Shiiki H, Dohi K (1998). Relationship between lupus nephritis activity and the serum level of soluble VCAM-1. Lupus..

[11] Molad Y, Miroshnik E, Sulkes J, Pitlik S, Weinberger A, Monselise Y (2002). Urinary soluble VCAM-1 in systemic lupus erythematosus: a clinical marker for monitoring disease activity and damage. Clin Exp Rheumatol..

[12] Wu T, Xie C, Wang HW, Zhou XJ, Schwartz N, Calixto S (2007). Elevated urinary VCAM-1, P-selectin, soluble TNF receptor-1, and CXC chemokine ligand 16 in multiple murine lupus strains and human lupus nephritis. J Immunol..

[13] Singh S, Wu T, Xie C, Vanarsa K, Han J, Mahajan T (2012). Urine VCAM-1 as a marker of renal pathology activity index in lupus nephritis. Arthritis Res Ther..

[14] Kaplanski G, Cacoub P, Farnarier C, Marin V, Gregoire R, Gatel A (2000). Increased soluble vascular cell adhesion molecule 1 concentrations in patients with primary or systemic lupus erythematosus-related antiphospholipid syndrome: correlations with the severity of thrombosis. Arthritis Rheum..

[15] Mueller SN, Germain RN (2009). Stromal cell contributions to the homeostasis and functionality of the immune system. Nat Rev Immunol..

[16] Cook-Mills JM, Marchese ME, Abdala-Valencia H (2011). Vascular cell adhesion molecule-1 expression and signaling during disease: regulation by reactive oxygen species and antioxidants. Antioxid Redox Signal..

[17] Koopman G, Keehnen RM, Lindhout E, Newman W, Shimizu Y, van Seventer GA (1994). Adhesion through the LFA-1 (CD11a/CD18)-ICAM-1 (CD54) and the VLA-4 (CD49d)-VCAM-1 (CD106) pathways prevents apoptosis of germinal center B cells. J Immunol..

[18] Levesque JP, Takamatsu Y, Nilsson SK, Haylock DN, Simmons PJ (2001). Vascular cell adhesion molecule-1 (CD106) is cleaved by neutrophil proteases in the bone marrow following hematopoietic progenitor cell mobilization by granulocyte colony-stimulating factor. Blood..

[19] Ulyanova T, Scott LM, Priestley GV, Jiang Y, Nakamoto B, Koni PA (2005). VCAM-1 expression in adult hematopoietic and nonhematopoietic cells is controlled by tissue-inductive signals and reflects their developmental origin. Blood..

[20] Tokoyoda K, Egawa T, Sugiyama T, Choi BI, Nagasawa T (2004). Cellular niches controlling B lymphocyte behavior within bone marrow during development. Immunity..

[21] Koni PA, Joshi SK, Temann UA, Olson D, Burkly L, Flavell RA (2001). Conditional vascular cell adhesion molecule 1 deletion in mice: impaired lymphocyte migration to bone marrow. J Exp Med..

[22] Leuker CE, Labow M, Muller W, Wagner N (2001). Neonatally induced inactivation of the vascular cell adhesion molecule 1 gene impairs B cell localization and T cell-dependent humoral immune response. J Exp Med..

[23] Hochberg MC. Updating the American College of Rheumatology revised criteria for the classification of systemic lupus erythematosus. Arthritis Rheum. 1997;40(9):1725. 10.1002/art.1780400928 9324032

[24] Ruperto N, Hanrahan LM, Alarcon GS, Belmont HM, Brey RL, Brunetta P (2011). International consensus for a definition of disease flare in lupus. Lupus..

[25] Bencivelli W, Vitali C, Isenberg DA, Smolen JS, Snaith ML, Sciuto M, et al. Disease activity in systemic lupus erythematosus: report of the Consensus Study Group of the European Workshop for Rheumatology Research. III. Development of a computerised clinical chart and its application to the comparison of different indices of disease activity. The European Consensus Study Group for Disease Activity in SLE. Clin Exp Rheumatol. 1992;10(5):549-54. 1458711

[26] American College of Rheumatology Ad Hoc Committee on Systemic Lupus Erythematosus Response Criteria (2004). The American College of Rheumatology response criteria for systemic lupus erythematosus clinical trials: measures of overall disease activity. Arthritis Rheum..

[27] Ridker PM (2007). C-reactive protein and the prediction of cardiovascular events among those at intermediate risk: moving an inflammatory hypothesis toward consensus. J Am Coll Cardiol..

[28] Jacobi AM, Odendahl M, Reiter K, Bruns A, Burmester GR, Radbruch A (2003). Correlation between circulating CD27high plasma cells and disease activity in patients with systemic lupus erythematosus. Arthritis Rheum..

[29] Jacobi AM, Reiter K, Mackay M, Aranow C, Hiepe F, Radbruch A (2008). Activated memory B cell subsets correlate with disease activity in systemic lupus erythematosus: delineation by expression of CD27, IgD, and CD95. Arthritis Rheum..

[30] Nawawi H, Osman NS, Annuar R, Khalid BA, Yusoff K (2003). Soluble intercellular adhesion molecule-1 and interleukin-6 levels reflect endothelial dysfunction in patients with primary hypercholesterolaemia treated with atorvastatin. Atherosclerosis..

[31] Franco SJ, Huttenlocher A (2005). Regulating cell migration: calpains make the cut. J Cell Sci..

[32] McHale JF, Harari OA, Marshall D, Haskard DO (1999). TNF-alpha and IL-1 sequentially induce endothelial ICAM-1 and VCAM-1 expression in MRL/lpr lupus-prone mice. J Immunol..

[33] Marshall D, Dangerfield JP, Bhatia VK, Larbi KY, Nourshargh S, Haskard DO (2003). MRL/lpr lupus-prone mice show exaggerated ICAM-1-dependent leucocyte adhesion and transendothelial migration in response to TNF-alpha. Rheumatology (Oxford).

[34] Norman MU, James WG, Hickey MJ (2008). Differential roles of ICAM-1 and VCAM-1 in leukocyte-endothelial cell interactions in skin and brain of MRL/faslpr mice. J Leukoc Biol..

[35] Garton KJ, Gough PJ, Philalay J, Wille PT, Blobel CP, Whitehead RH (2003). Stimulated shedding of vascular cell adhesion molecule 1 (VCAM-1) is mediated by tumor necrosis factor-alpha-converting enzyme (ADAM 17). J Biol Chem..

[36] Singh RJ, Mason JC, Lidington EA, Edwards DR, Nuttall RK, Khokha R (2005). Cytokine stimulated vascular cell adhesion molecule-1 (VCAM-1) ectodomain release is regulated by TIMP-3. Cardiovasc Res..

[37] Dreymueller D, Pruessmeyer J, Groth E, Ludwig A (2012). The role of ADAM-mediated shedding in vascular biology. Eur J Cell Biol..

[38] Koopman G, Parmentier HK, Schuurman HJ, Newman W, Meijer CJ, Pals ST (1991). Adhesion of human B cells to follicular dendritic cells involves both the lymphocyte function-associated antigen 1/intercellular adhesion molecule 1 and very late antigen 4/vascular cell adhesion molecule 1 pathways. J Exp Med..

[39] El Shikh ME, El Sayed R, Szakal AK, Tew JG (2006). Follicular dendritic cell (FDC)-FcgammaRIIB engagement via immune complexes induces the activated FDC phenotype associated with secondary follicle development. Eur J Immunol..

[40] Carrasco YR, Batista FD (2006). B-cell activation by membrane-bound antigens is facilitated by the interaction of VLA-4 with VCAM-1. EMBO J..

[41] Isenberg DA, Manson JJ, Ehrenstein MR, Rahman A (2007). Fifty years of anti-ds DNA antibodies: are we approaching journey’s end?. Rheumatology (Oxford).

