# Supplementary figures and images for: Improved monitoring of clinical response in Systemic Lupus Erythematosus by longitudinal trend in soluble vascular cell adhesion molecule-1
Source: Arthritis Res Ther. 2016 Jan 8;18:5. doi: 10.1186/s13075-015-0896-7 (PMC4718032; doi:10.1186/s13075-015-0896-7)

Supplementary Figure 1

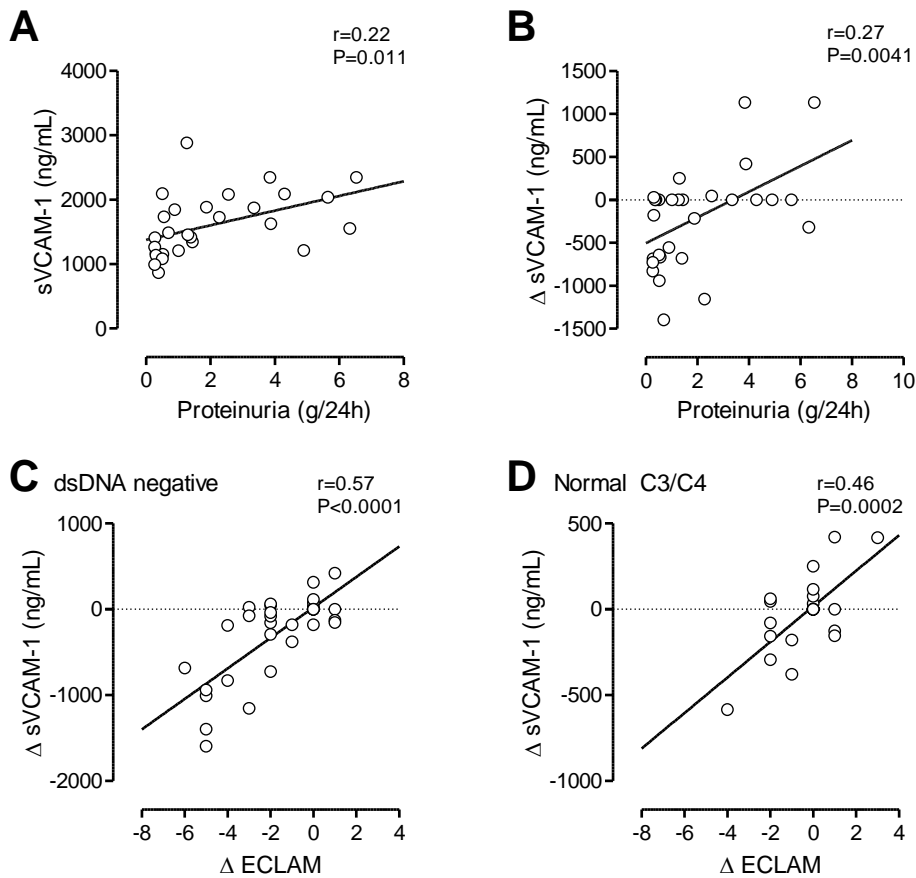

Supplement: Additional file 1: Figure S1. — Showing analysis of ∆sVCAM-1 levels in SLE subgroups. Plots showing correlation of A unadjusted sVCAM-1 and B ∆sVCAM-1 levels with proteinuria levels in individuals with lupus nephritis. Correlation of ∆sVCAM-1 with change in SLE disease activity measured by ∆ECLAM in C SLE individuals with negative dsDNA titres and D normocomplementaemic SLE individuals. (PDF 36 kb) [file 13075_2015_896_MOESM1_ESM.pdf]
